# A population‐based estimate of the economic burden of influenza in Peru, 2009–2010

**DOI:** 10.1111/irv.12357

**Published:** 2016-01-29

**Authors:** Yeny O. Tinoco, Eduardo Azziz‐Baumgartner, Hugo Rázuri, Matthew R. Kasper, Candice Romero, Ernesto Ortiz, Jorge Gomez, Marc‐Alain Widdowson, Timothy M. Uyeki, Robert H. Gilman, Daniel G. Bausch, Joel M. Montgomery, the Peru Influenza Cohorts Working Group, Giselle M. Soto, Maria E. Silva, Maria C. Guezala, Carlos Figueroa, Carolina Guevara, Erik Reaves, Eric S. Halsey, Maya Williams, Hector H. García, Armando E. González

**Affiliations:** ^1^ U.S. Naval Medical Research Unit No. 6CallaoPeru; ^2^ Johns Hopkins Bloomberg School of Public HealthBaltimoreMDUSA; ^3^ Influenza DivisionCenters for Disease Control and PreventionAtlantaGAUSA; ^4^ General Directorate of EpidemiologyMinistry of HealthLimaPeru; ^5^ Tulane School of Public Health and Tropical MedicineNew OrleansLAUSA; ^6^ Division of Global Disease Detection International Emerging Infections ProgramCenters for Disease Control and PreventionNairobiKenya

**Keywords:** Costs, healthcare economics, human influenza, population based, prevention and control

## Abstract

**Introduction:**

Influenza disease burden and economic impact data are needed to assess the potential value of interventions. Such information is limited from resource‐limited settings. We therefore studied the cost of influenza in Peru.

**Methods:**

We used data collected during June 2009–December 2010 from laboratory‐confirmed influenza cases identified through a household cohort in Peru. We determined the self‐reported direct and indirect costs of self‐treatment, outpatient care, emergency ward care, and hospitalizations through standardized questionnaires. We recorded costs accrued 15‐day from illness onset. Direct costs represented medication, consultation, diagnostic fees, and health‐related expenses such as transportation and phone calls. Indirect costs represented lost productivity during days of illness by both cases and caregivers. We estimated the annual economic cost and the impact of a case of influenza on a household.

**Results:**

There were 1321 confirmed influenza cases, of which 47% sought health care. Participants with confirmed influenza illness paid a median of $13 [interquartile range (IQR) 5–26] for self‐treatment, $19 (IQR 9–34) for ambulatory non‐medical attended illness, $29 (IQR 14–51) for ambulatory medical attended illness, and $171 (IQR 113–258) for hospitalizations. Overall, the projected national cost of an influenza illness was $83–$85 millions. Costs per influenza illness represented 14% of the monthly household income of the lowest income quartile (compared to 3% of the highest quartile).

**Conclusion:**

Influenza virus infection causes an important economic burden, particularly among the poorest families and those hospitalized. Prevention strategies such as annual influenza vaccination program targeting SAGE population at risk could reduce the overall economic impact of seasonal influenza.

## Introduction

Each year up to ~1 billion people may become ill with influenza virus,^1^ resulting in increased mortality, morbidity, and economic loss, not only among high‐risk groups but also among otherwise healthy persons.^2^, ^3^ Investigators have estimated both the direct (health‐related) and indirect (productivity loss) costs associated with influenza, although mostly in more developed temperate countries.^4^ The studies show that influenza illness can result in a large economic burden to ill individuals, their families, and healthcare providers. Studies conducted in the United States showed lost productivity from missed work among ill persons and caregivers to account for >50% of the total cost of an influenza episode.^5^

Most studies have focused on data from hospital discharge and healthcare utilization records in middle‐ or high‐income countries.^4^, ^5^ This approach excludes persons who did not seek medical care because of financial or logistical constraints. Furthermore, few estimates are available about the economic impact‐related caregivers' lost productivity and money spent on over‐the‐counter medications and other out‐of‐pocket expenses. Last, most data are from high‐income countries, despite the fact that influenza occurs in low‐ and middle‐income countries at similar or greater rates.^5^, ^6^, ^7^ To assess the full economic burden of influenza in a middle‐income country, we estimated both the direct and indirect costs among persons enrolled in a community‐based cohort study of influenza in Peru.

## Methods

### Study population

The study population consisted of participants with laboratory‐confirmed influenza detected during June 2009 through December 2010 through a population‐based cohort study conducted by U.S. Naval Medical Research Unit No. 6 with support from the Peruvian Ministry of Health, the U.S. Centers for Disease Control and Prevention (Atlanta, GA, USA), and the Armed Forces Health Surveillance Center (Silver Spring, MD, USA).^8^, ^9^ In the study, over 2000 randomly selected households comprising more than 7200 people in four geographically diverse sites in Peru were visited up to three times a week to screen household members for influenza‐like illness (ILI, defined as measured or subjective fever and sore throat or cough within the previous 2 days). Nasopharyngeal swabs were collected from identified cases and tested for influenza types A and B viruses by real‐time reverse transcription polymerase chain reaction.^8^

### Measurement of costs

The total cost of influenza was estimated through direct and indirect costs using a standardized questionnaire. Study field workers recorded disease progression, healthcare‐seeking behavior, and associated costs 3 times/week for up to 15 days after the onset of ILI.

Direct costs were defined as those incurred as a direct result of seeking medical care for the acute illness, including hospital and healthcare worker fees, diagnostic tests (e.g., blood tests, radiology studies), prescription and over‐the‐counter medications, and transportation and other related expenditures (e.g., phone calls, food, financial loans). We took into account out‐of‐pocket expenditures of participants who had either private health insurance or who participated in the Peruvian Comprehensive Health Insurance (SIS).^10^ We also recorded out‐of‐pocket expenditures related to healthcare delivery through informal providers and methods, including pharmacy consultations, traditional healers, and herbal remedies. Field workers verified healthcare‐related costs from receipts when these were available.

Indirect costs were defined as lost wages resulting from absenteeism from productive activities as a result of influenza on the part of the ill person and/or their parent or guardian (hereafter referred to as caregivers). Costs and productivity loss were evaluated as total days lost from: (i) work, (ii) unpaid activities (e.g., homemakers and household chores), or (iii) school. To measure total productivity lost, we used a previously described method in which, at each household visit, the ill person or a household informant reported lost days of work or school and reduced productivity by assigning a daily index of lost days: 0 for an uninterrupted work day, 0·25 for partially disturbed or interrupted work day, 0·5 for partial work day, and 1 for a complete work day lost or total loss of productivity for the day.^11^

To accord monetary value to the time and productivity lost, we asked the ill person and/or caregiver to estimate the wages lost. For unpaid activities among participants aged ≥18 years, we multiplied the total lost days by the concurrent Peruvian monthly minimum wage divided by the 22 working days in a month. We recorded costs in Peruvian Nuevo Soles and then converted these to US$ after accounting for inflation and cost of dollar adjustments for 2009 as estimated by the National Bank of Peru.^12^ The total cost of each case of influenza was calculated as the sum of direct and indirect expenditures.

We explored the associations between cost and gender, age group (<5, 5–17, 18–49, 50–64, and ≥65 years), risk of complications (high versus low based on self‐reported and/or physician‐diagnosed comorbid conditions such as asthma, diabetes, cancer, chronic bronchitis, hypertension, and heart disease), and presence of complications (e.g., pneumonia and otitis media^13^). We also explored the associations between costs and the type of healthcare provider sought by participants based on five mutually exclusive healthcare‐seeking behaviors: (i) did not seek care; (ii) sought non‐medical attention, such as from traditional healers, drug stores, or family members; (iii) sought outpatient medical care; (iv) presented to an emergency room; or (v) was hospitalized.^14^ Each event was assigned to the highest level of medical care received in the 15 days of follow‐up. We assumed that each higher level of care received also included the costs of the lower levels of care.

There are five subsystems which provide health care in Peru: (i) The Ministry of Health that offers Comprehensive Health Insurance; (ii) Health Social Security (EsSalud) administered by the Ministry of Labour; (iii) Armed forces health assistance, administered by the Ministry of Defense; (iv) National Police health assistance administered by the Interior Ministry; and (v) private insurance companies.^15^, ^16^ We classified each event according to the health insurance utilization status: (i) no utilization, (ii) Comprehensive Health Insurance which is intended for more vulnerable population (people without any health insurance), and (iii) private and public insurance understood as a system where people contribute to pay for healthcare benefits from their salaries.

### Affordability assessment and distribution of costs across households

To assess participants' ability to afford the medical care they sought, direct, indirect, and total costs incurred were converted to a percentage of their reported monthly household income. We assigned a direct expenditure threshold of ≥10% of household monthly income as representative of a significant financial impact.^17^ To describe the distribution of cost for influenza episodes across households, we divided the cost of an influenza episode by the average monthly income reported by the family and stratified the analysis by the household income quartile: low (<$300), low–middle ($300–500), high–middle ($500–1000), and high (>$1000).

### Statistical analysis

Data from the four study sites were analyzed using the stata 12 statistical software package (Stata Corporation, College Station, TX, USA). The Kruskal–Wallis and Wilcoxon–Mann–Whitney tests were used as appropriate to determine group differences in the median direct, indirect, and total costs and number of days lost. We used generalized linear models with gamma family, log link, and cluster adjustment by participant to evaluate the association between costs and the aforementioned variable as previously described.^18^, ^19^ Variables that were significant (*P* < 0·05) in bivariate analysis were included in the multivariable analysis. The relative cost ratio represents the percentage change in each of the evaluated groups compared to the reference group. The reference group (designated as ‘Ref' in Table 3) is defined as having 100% of the cost, and the cost for each of the evaluated groups is expressed relative to the reference group. To estimate the annual economic burden of influenza for Peru, we multiplied influenza rates^7^ by the age‐stratified census population of Peru classified by the median total cost of an influenza episode per year.

### Institutional review board approval

This study was approved by the U.S. Naval Medical Research Unit No. 6 Ethics Committee (NMRCD.2009.005).

## Results

### Demographic and care‐seeking characteristics

During June 2009 to July December 2010, a total of 1321 cases of laboratory‐confirmed influenza illness were identified among 1215 participants in 730 households. Cases had a mean age of 16 years (range 1 month–91 years) and 51% was female (Table 1). The mean monthly household income was $648 (range $82–$4429). Approximately half (*n* = 643, 49%) of the influenza episodes occurred among school‐aged children aged 5–17 years. Approximately half of participants (*n* = 696, 53%) did not seek care from a medical provider. Eleven participants were hospitalized for a median of 4 days as a result of their influenza illness. Their households had to borrow money to pay for hospitalization expenses. We documented one death due to influenza A(H1N1)pdm09 during 2009. This death was excluded from the analysis because it will not provide enough information to understand the cost associated with deaths. Most persons (*n* = 1001, 76%) did not use health insurance and thus paid out‐of‐pocket for all direct illness‐related costs. One hundred and seventy‐eight (13%) were at high risk for complications from influenza illness (e.g., chronic cardiovascular or respiratory comorbidity)^20^; 28 (16%) of these developed complications such as pneumonia and otitis media. Complications also occurred among 89 (7%) participants who were not identified *a priori* as high risk.

**Table 1:** Demographic and care‐seeking characteristics of the study population with influenza

Influenza laboratory‐confirmed cases	No. of cases (%)
*N*	1321
Female	680 (51)
Year	
2009^a^	463 (35)
2010	858 (65)
Age	
<5 years	298 (23)
5–17 years	643 (49)
18–49 years	313 (24)
50–64 years	52 (4)
65 years+	15 (1)
Risk for complication^b^	
High	178 (13)
Developed complication^c^	
Yes	89 (7)
Level of medical care	
Self‐care alone	345 (26)
Seek non‐medical attention	351 (27)
Outpatients	575 (44)
Emergency ward	39 (3)
Hospitalized	11 (1)
Health insurance utilization	
No utilization	1001 (76)
Comprehensive Health Insurance	212 (16)
Private and public insurance	108 (8)
Monthly household income US$	
<$300	296 (22)
$300–<$500	264 (20)
$500–<$1000	346 (26)
≥$1000	202 (15)
Refused, unknown, missing	213 (16)

a 2009: June–December.

b Self‐reported, physician‐diagnosed chronic diseases: asthma, chronic bronchitis, tuberculosis, diabetes, cancer, hypertension, and heart diseases.

c Complications (e.g., pneumonia and otitis media).

### Costs

Most persons with influenza illness (*n *= 1041, 79%) incurred expense (Tabe S1). The total median cost to patients' families for influenza episodes was $22 [interquartile range (IQR) $31], of which $3 was direct (IQR 8) and $15 indirect (IQR 25) (Table 2). Medications accounted for the largest proportion of direct costs (63%), followed by transportation and other costs (17%), physician fees (15%), and diagnostic tests (4%) (Table 2). Total indirect costs were consistently higher than direct costs for all levels of medical care sought and were significantly higher among cases (median $6, IQR 21) than among caregivers (median $2; IQR 13) (*P *< 0·0005). The highest cost was observed among hospitalized persons (median $171; IQR 145) and the lowest among those who cared for themselves (median $13; IQR 21).

**Table 2:**
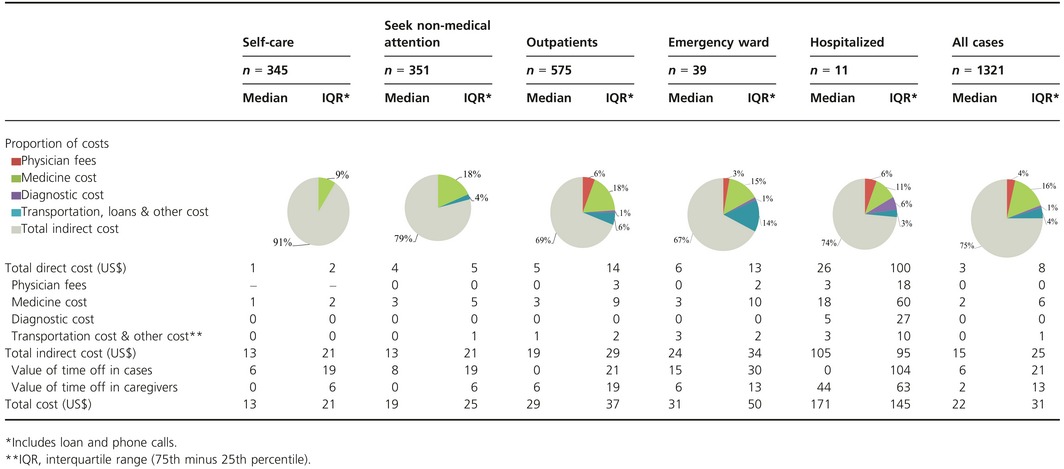
Cost of case of influenza by level of medical care in 2009 US$

On multivariable analysis, children aged <5 years had significantly higher direct costs, while their indirect costs were significantly lower than those of other age groups (Table 3). The indirect cost among females was 20% lower than among males (*P* < 0·01). Participants who developed complications (e.g., pneumonia and otitis media) associated with influenza had 100% increase in the direct costs over those who did not develop any complications (*P* < 0·001). Participants who had health insurance spent significantly (*P* < 0·001) less in direct out‐of‐pocket costs than participants who did not (70% less among participants who utilized Comprehensive Health Insurance and 50% less among participants insurance compared with participants without health insurance). Although the total cost for the lowest quartile households was significantly lower than for those in the highest quartile ($28 versus $36, respectively, *P* = 0·04), expenses comprised a significantly higher proportion of lowest quartile households' income (14% versus 3%, *P* < 0·001) (Table 4).

**Table 3:** Multivariate analysis of costs by various demographic and care‐seeking characteristics

Independent variables	Direct cost			Indirect cost			Total cost		
	Relative cost ratio^a^	95% Confidence intervals	*P* value	Relative cost ratio^a^	95% Confidence intervals	*P* value	Relative cost ratio^a^	95% Confidence intervals	*P* value
Age group									
<5 years	Ref			Ref			Ref		
5–17 years	0·7	0·6–0·9	<0·01	1·5	1·3–1·8	0·00	1·3	1·1–1·4	0·00
18–49 years	0·7	0·6–0·9	<0·01	2·4	2·0–2·9	0·00	1·8	1·5–2·1	0·00
50–64 years	0·6	0·4–0·9	0·03	1·6	1·3–2·2	0·00	1·3	1·0–1·6	0·03
65+ years	0·7	0·3–1·8	0·50	3·0	1·8–5·3	0·00	2·1	1·2–3·8	0·01
Gender									
Male	Ref			Ref			Ref		
Female	0·9	0·8–1·1	0·48	0·8	0·7–0·9	0·00	0·9	0·8–0·9	0·00
Risk for complication									
Non‐high risk	Ref			Ref			Ref		
High risk	1·2	1·0–1·5	0·10	1·2	1·0–1·4	0·07	1·2	1·0–1·4	0·02
Developed complications									
No	Ref			Ref			Ref		
Yes	2·0	1·3–2·9	<0·01	1·3	1·1–1·6	0·00	1·4	1·2–1·7	0·00
Health insurance utilization by provider									
No utilization	Ref			Ref			Ref		
Comprehensive Health Insurance	0·3	0·2–0·4	<0·01	0·9	0·7–1·1	0·16	0·6	0·5–0·8	0·00
Private and public insurance	0·5	0·3–0·7	<0·01	1·0	0·8–1·2	0·70	0·8	0·6–1·0	0·03
Level of medical care									
Self‐care	Ref			Ref			Ref		
Seek non‐medical attention	3·2	2·7–3·9	<0·01	1·2	1·0–1·4	0·06	1·4	1·2–1·6	0·00
Outpatient	8·6	7·0–10·4	<0·01	1·7	1·4–2·0	0·00	2·4	2·1–2·8	0·00
Emergency ward	14·9	7·0–31·5	<0·01	2·1	1·4–3·2	0·00	3·2	2·0–5·1	0·00
Hospitalized	45·6	20·4–102·2	<0·01	8·8	5·0–15·7	0·00	12·7	7·2–22·3	0·00

a Multivariate analysis, adjusting for age, gender, risk for complications, healthcare provider, development of complications, and level of medical care. Constant coefficients: 0·88 (direct cost), 2·56 (indirect cost), and 2·84 (total cost), accounts for children under 5 years, male, without risk for complications, did not develop complications, non‐health insurance utilization, and had self‐care.

**Table 4:** Mean costs per case^a^ of influenza by household income

Monthly household income US$^b^	No of HH^c^ (%)	Direct cost US$ (% of the HH income)	Indirect cost US$ (% of the HH income)	Total cost US$ (% of the HH income)
Lowest quartile (<$300)	146 (20·0)	5·5 (2·7)	22·8 (11·4)	28·3 (14·1)
Low–middle quartile ($300–<$500)	125 (17·1)	7·0 (1·9)	22·9 (6·2)	30·0 (8·1)
High–middle quartile ($500–<$1000)	209 (28·6)	8·7 (1·3)	26·3 (3·9)	35·1 (5·2)
Highest quartile (≥$1000)	115 (15·8)	9·6 (0·7)	26·1 (2·0)	35·7 (2·6)
Refused, unknown, missing	135 (18·5)	10·0 (NA)	24·7 (NA)	35·7 (NA)
Overall	730 (100)	8·2 (1·7)	24·6 (6·1)	32·7 (7·8)

a Represents the over mean or all 1321 laboratory‐confirmed influenza cases.

b Categories based on interquartile ranges (25th, 50th, 75th percentile).

c HH, household.

### Lost productivity from missed work and school

Seventy‐one percent (*n* = 942) of 1321 persons with influenza reported days lost from work, school, or routine unpaid activities (Table S1). Caregivers also took time off during 52% (*n* = 677) of illness events. Although the median number of days of productive work lost was higher for paid workers and their caregivers (4 days lost for the ill person and 2 for the caregivers) compared to those engaged in unpaid labor (2 days lost for the ill person and 1 for the caregivers), this difference was not statistically significant (Table 5). School‐aged children missed a mean of 3 school days (median 2; IQR 3) per episode of influenza.

**Table 5:** Days lost from school, work, and unpaid activities in persons with influenza and caregivers by level of medical care

Days lost	Self‐care		Seek non‐medical attention		Outpatients		Emergency ward		Hospitalized		*P* value	All cases	
	Median	IQR^a^	Median	IQR^a^	Median	IQR^a^	Median	IQR^a^	Median	IQR^a^		Median	IQR^a^
Influenza case													
School^b^	1·8	2	1·8	2	2·6	2·3	2	3	9·0	–	0·00	2·0	2·5
Work place	2·6	3	2·8	2·8	4	4	4·8	–	9·4	–	0·03	3·5	3
Unpaid activity	1·3	1·13	1·5	1·3	1·8	1·9	3	0·8	12	–	0·00	1·8	1·8
Caregiver													
Work place	1·5	1·5	2·5	2·3	2·3	1·5	5	4·6	2·8	–	0·60	2·3	2·5
Unpaid activity	0·8	0·75	0·8	0·8	1	1·25	1	0·3	3·8	4·8	0·00	1	1

a IQR, interquartile range (75th minus 25th percentile).

b School‐aged children: 5–17 years old.

### Projected national costs of influenza

Using overall influenza rates within the cohort (Table 6),^7^ the median costs from this study (Table 2) and the Peruvian census, we estimated an annual cost of influenza illness of $83 million in 2009 and $85 million in 2010 (Table 6). Influenza‐associated cost represented approximately 0·06–0·07% of gross domestic product.^21^

**Table 6:** Estimated annual economic burden of influenza by age group in Peru in 2009–2010 (2009 US$)

Year and age group	Annual incidence of influenza A and B^a^	Direct cost		Indirect cost		Total cost			
		Median cost	National (millions)	Median cost	National (millions)	Median cost	National (millions)	% of total	% of GDP
2009			14·4		39·4		83·1		0·07
<5 years	325	4·2	3·7	12·5	11·1	24·0	21·3	25·6	
5–17 years	305	3·3	7·4	0·3	0·7	12·5	27·7	33·4	
18–49 years	62	3·7	2·9	26·6	21·1	33·4	26·5	31·9	
50–64 years	35	4·6	0·4	34·4	3·3	45·0	4·4	5·3	
65 years+	17	0·9	0·0	106·3	3·2	107·1	3·2	3·9	
2010			11·1		66·3		85·2		0·06
<5 years	277	5·2	3·9	8·6	6·5	17·2	13·0	15·2	
5–17 years	225	2·7	4·5	23·6	38·7	27·3	44·8	54·0	
18–49 years	74	2·3	2·2	17·2	16·4	21·8	20·8	25·1	
50–64 years	65	1·4	0·3	16·1	2·9	20·1	3·6	4·4	
65 years+	46	3·3	0·3	21·4	1·7	35·9	2·9	3·5	

Peruvian population: <5 years: 2 724 620; 5–17 years: 7 288 110; 18–49 years: 12 858 994; 50–64 years: 2 775 746; and 65 years+: 1 764 687.

a Influenza incidence rates: events per 1000 person‐year.

## Discussion

Our study demonstrates the significant economic impact of influenza in Peru, a burden imposed among ill person and their families, groups for whom the costs are often underreported.^14^ The burden was particularly heavy on the poorest families, for whom the cost of a single episode of influenza in a family member typically represented more than a tenth of the total household monthly income. The magnitude of the direct costs noted in our study was similar to that found in other low‐ and middle‐income countries among non‐hospitalized (range $4–16) and hospitalized (range $60–575) persons^22^, ^23^, ^24^ and lower than the cost among hospitalized patients in high‐income countries (range $5402–6124).^4^, ^25^, ^26^

We were able to document the costs incurred among persons who did not seek traditional medical care at healthcare centers because our participants were identified through population‐based surveillance.^7^ Our study demonstrates that more than half of laboratory‐confirmed influenza cases incurred costs when self‐medicating and not when seeking care from a clinician. These costs are typically missed from studies that only quantify costs associated with medically attended acute respiratory infections.

Our population‐based surveillance platform also allowed us to estimate indirect costs incurred by households as a result of influenza illness. Indirect costs represented a substantive portion of the total costs.

The mean number of work days lost because of influenza in our study was similar to that reported from other countries such as France, Finland, Italy, Japan, and the United States.^11^, ^27^, ^28^, ^29^, ^30^ Our results were in keeping with various studies that suggest that indirect costs comprise the bulk of the economic burden associated with influenza illness in middle‐ and high‐income countries.^5^, ^31^, ^32^ Exceptions are occasionally noted in very low‐income countries, such as Bangladesh, where the extremely low remuneration rates for lost work and productivity deflate indirect costs relative to direct costs.^24^ Additionally, we documented a lower indirect cost for female. This may be explained by lower salary for women compared with salary for men when paid activities are executed.^33^ Moreover, this finding may also be explained by many women engaging in unpaid activities such as household chores or childcare. We also documented substantive school absenteeism associated with decreased scholastic performance among children missing school.^24^, ^34^

We note various limitations to our study: first, our data were based on self‐reported information when receipts were missing and could have resulted in inaccurate reporting of costs because of incomplete recall. However, recall errors were likely minimal considering that we visited households prospectively every other day and corroborated costs with receipts. Second, we could have underestimated indirect costs because we did not collect data about the time spent traveling to health facilities or waiting and receiving treatment at healthcare services. Third, we did not take into account the costs to the insurance provider, again leading to underestimation of the total costs to the society. Providers typically earn their income from persons whose health care is subsidized.^15^ Finally, we did not scale cost by severity of symptoms.

Our data demonstrate that influenza costs Peru tens of millions of dollars annually. Peru currently vaccinates against influenza illness, but coverage among the general population is modest (5%).^35^ Vaccine impact models could help health officials explore the potential value increasing influenza vaccination coverage especially among World Health Organization‐recommended populations.^36^ The potential value of non‐pharmaceutical interventions such as hand washing, enhanced respiratory hygiene campaigns, and school‐based interventions^37^ should also be explored.

## Disclaimers

The views expressed in this article are those of the author and do not necessarily reflect the official policy or position of the Department of the Navy, Department of the Army, Department of Defense, Centers for Disease Control and Prevention, nor the U.S. Government.

Several authors of this manuscript are military service members or employees of the U.S. government. This work was prepared as part of their official duties. Title 17 U.S.C. §105 provides that ‘Copyright protection under this title is not available for any work of the United States Government.' Title 17 U.S.C. §101 defines a U.S. Government work as a work prepared by a military service member or employee of the U.S. Government as part of that person's official duties. The authors declare that they have no competing interests.

## Supporting information


**Table S1.** Distribution of influenza laboratory‐confirmed cases by level of medical care and cost components.

## Peru Influenza Cohorts Working Group

Peru Influenza Cohorts Working Group: Giselle M. Soto, Maria E. Silva, Maria C. Guezala, Carlos Figueroa, Carolina Guevara, Erik Reaves, Eric S. Halsey, Maya Williams, Hector H. García, Armando E. González.
