# Supplementary material for: A population‐based estimate of the economic burden of influenza in Peru, 2009–2010
Source: Influenza Other Respir Viruses. 2016 Jan 29;10(4):301–9. doi: 10.1111/irv.12357 (PMC4910177; doi:10.1111/irv.12357)
Supplement: Supplementary file 1 — Table S1. Distribution of influenza laboratory‐confirmed cases by level of medical care and cost components. [file IRV-10-301-s001.docx]

**Supplement 1.** Distribution of influenza laboratory-confirmed cases by level of medical care and cost components

| **Cost components** | **Self-care** | **Seek non- medical attention** | **Outpatients** | **Emergency ward** | **Hospitalized** | **Total cases** |
| --- | --- | --- | --- | --- | --- | --- |
|  | **n (%)** | **n (%)** | **n (%)** | **n (%)** | **n (%)** | **n (%)** |
| Total number of influenza cases | 345 | 351 | 575 | 39 | 11 | 1321 |
| Cases reporting expenses for ^a^ |  |  |  |  |  |  |
| Physician fees | 0(0) | 5 (1) | 266 (46) | 17 (44) | 8 (73) | 296 (22) |
| Medicine cost | 219 (63) | 340 (97) | 440 (77) | 32 (82) | 10 (91) | 1041 (79) |
| Diagnostic cost | 2 (1) | 5 (1) | 32 (6) | 4 (10) | 6 (55) | 49 (4) |
| Transportation cost | 2 (1) | 139 (40) | 347 (60) | 35 (90) | 11 (100) | 534 (40) |
| Loans | 4 (1) | 6 (2) | 40 (7) | 2 (5) | 11 (100) | 52 (4) |
|  |  |  |  |  |  |  |
| Cases reporting time off |  |  |  |  |  |  |
| From school ^b^ | 153 (44) | 160 (46) | 254 (44) | 17 (44) | 2 (18) | 586 (44) |
| From work | 14 (4) | 29 (8) | 28 (5) | 2 (5) | 2 (18) | 75 (6) |
| From unpaid activity | 88 (26) | 87 (25) | 96 (17) | 7 (18) | 3 (27) | 281 (21) |
|  |  |  |  |  |  |  |
| Caregivers reporting time off |  |  |  |  |  |  |
| From work | 11 (3) | 15 (4) | 49 (9) | 9 (23) | 3 (27) | 87 (7) |
| From unpaid activity | 132 (38) | 129 (37) | 303 (53) | 19 (49) | 7 (64) | 590 (45) |

^a^ Percentage may not add 100% because one same episode reported more than one expenditure

^b^ School age children: 5-17 years old, children under 5 years were not considered for time off calculation
